# Effects of nanosized water droplet generation on number concentration measurement of virus aerosols when using an airblast atomizer

**DOI:** 10.1038/s41598-022-10440-4

**Published:** 2022-04-21

**Authors:** Milad Massoudifarid, Amin Piri, Jungho Hwang

**Affiliations:** Department of Mechanical Engineering, Yonsei University, Seoul, 03722 Republic of Korea

**Keywords:** Mechanical engineering, Optical techniques, Environmental sciences, Engineering, Biological techniques, Biophysical methods, Microbiology techniques

## Abstract

Development of efficient virus aerosol monitoring and removal devices requires aerosolization of the test virus using atomizers. The number concentration and size measurements of aerosolized virus particles are required to evaluate the performance of the devices. Although diffusion dryers can remove water droplets generated using atomizers, they often fail to remove them entirely from the air stream. Consequently, particle measurement devices, such as scanning mobility particle sizer (SMPS), can falsely identify the remaining nanosized water droplets as virus aerosol particles. This in turn affects the accuracy of the evaluation of devices for sampling or removing virus aerosol particles. In this study, a plaque-forming assay combined with SMPS measurement was used to evaluate sufficient drying conditions. We proposed an empirical equation to determine the total number concentration of aerosolized particles measured using the SMPS as a function of the carrier air flow rate and residence time of the particles in the diffusion dryers. The difference in the total number concentration of particles under sufficient and insufficient diffusion drying conditions was presented as a percentage of error.

## Introduction

Virus aerosol exposure is associated with adverse health effects. Characterizing and quantifying airborne pathogens is crucial to exposure and risk assessments. Various aerosol instrument systems, such as air samplers and antimicrobial filtering devices, have been developed^1,2^. Developing new devices and techniques for applications in an ambient environment requires numerous tests and accurate device performance evaluations in controlled environments.

An airblast atomizer aerosolizes virus particles suspended in a liquid solution. When compressed air enters the atomizer, the pressure from the air jet promotes aerosolization of the water droplets containing virus particles^3^. The air flow rate (AFR), particle concentration in the liquid solution of the atomizer, and geometry of the atomizer affect aerosolized particle generation rate^4^. Since most aerosol instruments are sensitive to moisture, a series of diffusion dryers consisting of a circular tube with absorbing walls surrounded by silica-gel desiccant beads are used to remove moisture. Excess moisture within an aerosol moves into the dry environment due to diffusion. This leads to dehumidification, which is an adsorption process that results from the pressure difference of water vapor between the desiccant surface and surrounding air^5,6^. Hence, the residence time, dimensions of the dryer, and AFR, which is utilized in optimizing aerosol drying^7,8^ govern the design of a diffusion dryer.

During aerosolization experiments, the aerosol number concentration is measured using a particle measurement device, such as a scanning mobility particle sizer (SMPS). If the diffusion dryers do not entirely remove moisture from the airstream^6,9,10^, then SMPS data can be incorrectly interpreted and used in calculations. Inadequate moisture removal interferes with the measurement of virus aerosol particles if the moisture particles are within the same size range as the pure water droplets generated.

The size distribution and total number concentration of aerosol particles generated from solutions with and without virus particles were measured for different AFRs and residence times (τ) in the diffusion dryers. Plaque-forming assay data and SMPS data were used to determine sufficient drying conditions. The difference in the total number concentrations of aerosolized particles at sufficient and insufficient drying conditions was presented as a percentage of the error. Further, we proposed an empirical equation to determine total number concentration of aerosolized particles.

## Results

### Effect of air flow rate and residence time on size distribution and number concentration

**Figure 1:**
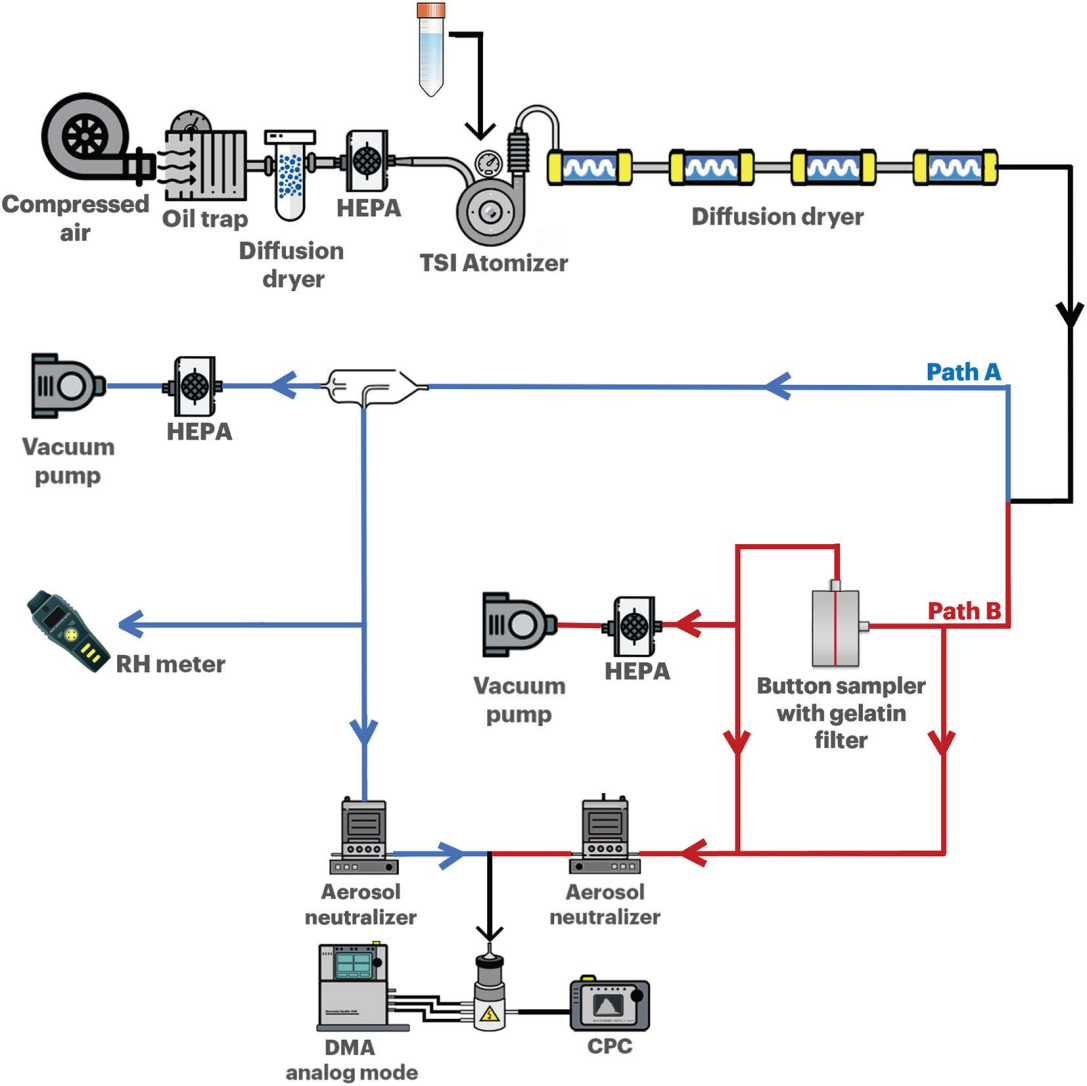
Schematic of the experimental design used to generate aerosol particles.

Experiments were performed using an atomizer solution without virus particles. Path A of Fig. 1 was utilized in the experiments. Details of the experimental devices are presented in “Methods” section. The variables for the different types of diffusion dryers are listed in Table 1.

**Table 1:** Characteristics of different types of diffusion dryers used during experiments.

Diffusion dryer type	Tube diameter *w*_*i*_ (m)	Tube length *L*_*i*_ (m)
*D*_*A*_	0.02	0.28
*D*_*B*_	0.02	0.24
*D*_*C*_	0.015	0.48

The residence time, τ, of the air flow in the diffusion dryers calculated using the following equation:

1 $$\uptau =\sum _{{\rm i}=0}^{{\rm i}=\mathrm{n}}\frac{\uppi {\mathrm{w}}_{{\rm i}}^{2}{\mathrm{L}}_{{\rm i}}}{4{\upgamma {\rm Q}}}$$

where n denotes the number of diffusion dryers, ${\mathrm{w}}_{{\rm i}}$ denotes the tube diameter of the ${\mathrm{i}}_{{\rm th}}$ diffusion dryer, ${\mathrm{L}}_{{\rm i}}$ denotes length of the ${\mathrm{i}}_{{\rm th}}$ diffusion dryer, Q is the AFR (2 to 5 L/min), and $\upgamma$ is a constant corresponding to 1.67 × 10^–5^ (min m^3^/L s).

**Figure 2:**
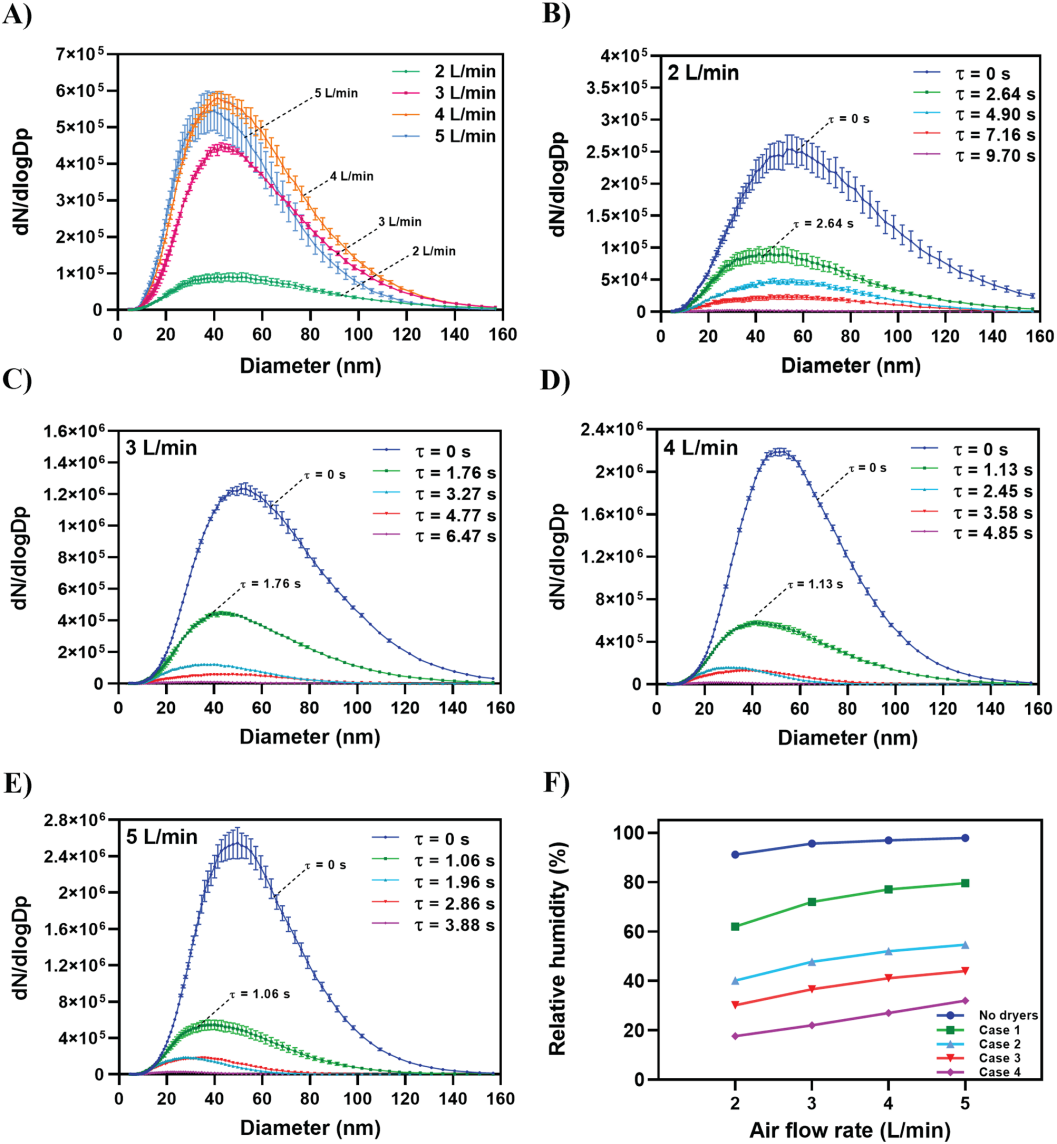
Effects of air flow rate and residence time in diffusion dryers on number concentration and size distribution of the aerosolized water droplets. (**A**) Case 1: one diffusion dryer is used (Table 2). (**B**) Air flow rate is 2 L/min, and residence time of 2.64, 4.90, 7.16, and 9.70 s represent Case 1, Case 2, Case 3, and Case 4, respectively (Table 2). (**C**) Air flow rate is 3 L/min, and residence time of 1.76, 3.27, 4.77, and 6.47 s represent Case 1, Case 2, Case 3, and Case 4, respectively (Table 2). (**D**) Air flow rate is 4 L/min, and residence time of 1.13, 2.45, 3.58, and 4.85 s represent Case 1, Case 2, Case 3, and Case 4, respectively (Table 2). (**E**) Air flow rate is 5 L/min, and residence time of 1.06, 1.96, 2.86, and 3.88 s represent Case 1, Case 2, Case 3, and Case 4, respectively (Table 2). (**F**) Effect of the air flow rate on RH is shown for each case.

Figure 2A shows the number concentrations and size distributions of the aerosolized water droplets at different AFRs when using one diffusion dryer (Case 1, Table 2). The water droplet size ranged from 4 to 160 nm. When AFR increased, the residence time decreased, and the droplet number concentration increased. Figure 2B, C, D, and E shows the effects of the residence time in the diffusion dryers (Table 2) at constant AFRs corresponding to 2, 3, 4, and 5 L/min, respectively. The original water droplet size distributions right after the air stream exiting the atomizer (τ = 0) were measured. The results are shown in Fig. 2B, C, D and E. The results indicate that the concentration of aerosolized water droplets decreased as the residence time increased. Figure 2F shows that the relative humidity (RH) decreased by 10% when the AFR decreased from 5 to 2 L/min for all cases. At a constant flow rate, the RH decreased by approximately 70% in Case 4.

**Table 2:** Overview of different dryer combinations, air flow rates, and corresponding residence times.

Experiment case	Case 1	Case 2	Case 3	Case 4
Combination of diffusion dryers	${D}_{A}$	${D}_{A}+{D}_{B}$	${D}_{A}+{2D}_{B}$	${D}_{A}+{2D}_{B}$+${D}_{C}$
Air flow rate (L/min)	**Residence time, **${\varvec{\tau}}\boldsymbol{ }({\varvec{s}})$			
2	2.64	4.9	7.16	9.7
3	1.76	3.27	4.77	6.47
4	1.13	2.45	3.58	4.85
5	1.06	1.96	2.86	3.88

The details of the results in Fig. 2 are presented in Table S1 in Supplementary Materials. For example, when an AFR of 2 L/min was used, applying different residence times reduced the total droplet number concentrations to 5.9 × 10^4^ cm^–3^ (τ = 2.64 s), 3 × 10^4^ cm^–3^ (τ = 4.90 s), and 1.6 × 10^4^ cm^–3^ (τ = 7.16 s), which correspond to 60%, 80%, and 89% decrease in total number concentration, respectively. Table S1 shows the changes in the total number concentration of generated droplets for different residence times and flow rates. The results indicated that the total droplet number concentration exponentially decreases when residence time increases for all tested flow rates.

### Total number concentration as an exponential decay regression

Conversely, for each case (Table 2), the total number concentration increased with increases in the flow rate. Thus, the total number concentration of droplets (${\mathrm{N}}_{{\rm t}}$) can be described as an exponential decay function of the residence time ($\tau$) as follows:

2 $${\mathrm{N}}_{{\rm t}}(\uptau )={\mathrm{N}}_{0}\mathrm{exp}(-\mathrm{k\tau })$$

where ${\mathrm{N}}_{0}$ and k denote the total number concentration of droplets at τ = 0 (cm^–3^) and decay constant (1/s), respectively; both are linearly proportional to the flow rate:

3 $${\mathrm{N}}_{0}={\upalpha {\rm Q}}$$

4 $$\mathrm{k}={\upbeta {\rm Q}}$$

where Q denotes the flow rate (L/min). The constants $\alpha$ and $\beta$ correspond to 2.31 × 10^5^ min/L cm^3^ and 0.251 min/L s, respectively. The results are listed in Table 3.

**Table 3:** Values of ${N}_{0}$ and k coefficients and R^2^ for the non-linear, exponential decay regression $[{N}_{t}(\tau )={N}_{0}exp(-k\tau )]$.

Flow rate (L/min)	2	3	4	5
*N*_0_ (cm^−3^)	1.492e + 5	6.425e + 5	9.997e + 5	1.15e + 6
*k* (1/s)	0.3774	0.6007	0.9703	1.1219
R^2^	0.99	0.99	0.99	0.99

### Optimal residence time in diffusion dryers for maximizing droplet removal

**Figure 3:**
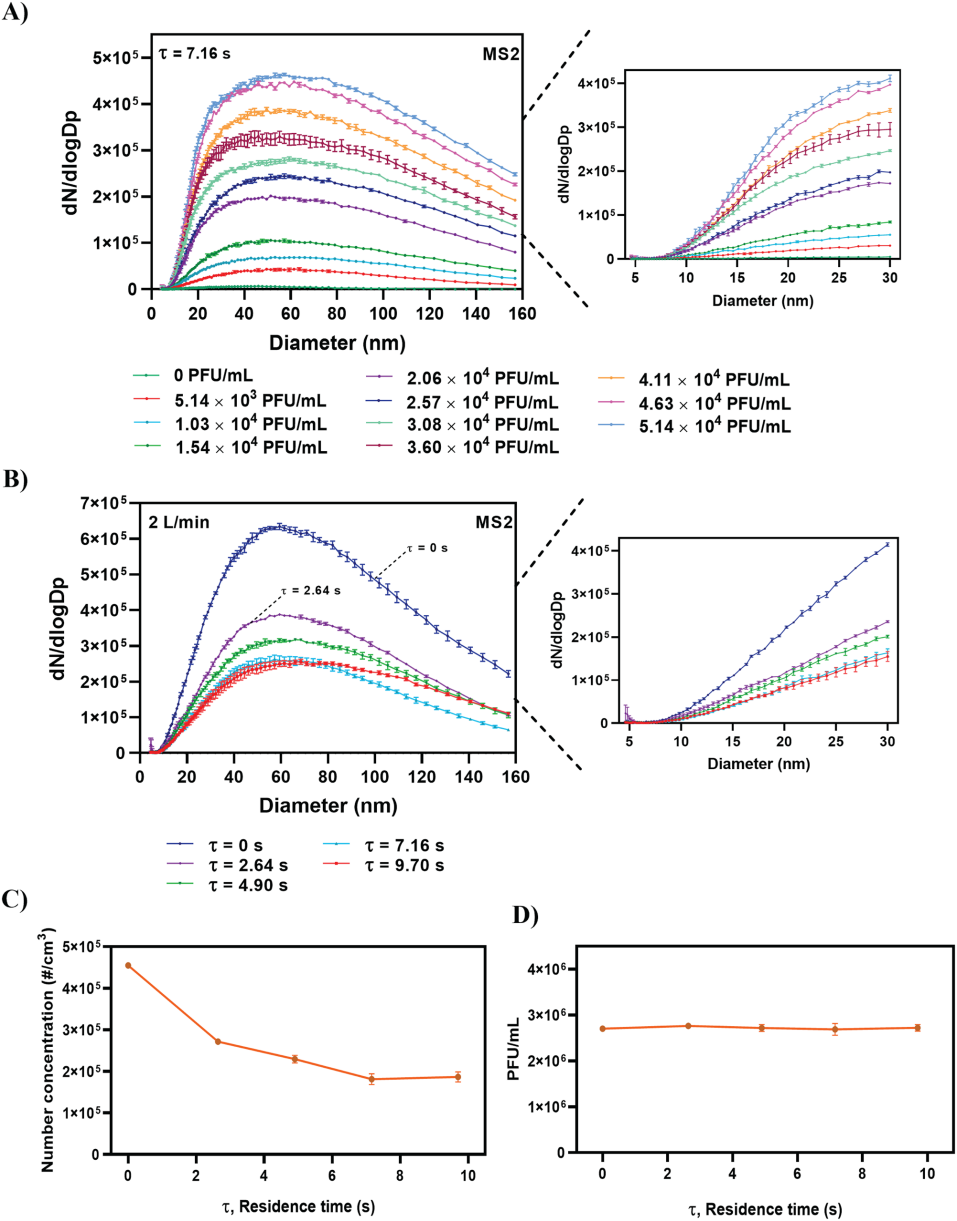
Effects of different MS2 virus solution concentrations and residence times on number concentration and size distribution of the aerosolized MS2 bacteriophage. (**A**) Effect of different MS2 virus solution concentrations on size distribution and concentration of the aerosolized particles at $\tau$ = 7.16 s (Table 2). (**B**) Effect of different residence times on size distribution and concentration of the aerosolized particles under a constant air flow rate of 2 L/min. (**C**) Effects of different residence times on total number concentration of aerosolized MS2 virus. (**D**) Plaque analysis results for the sampled MS2 bacteriophage virus under different residence times.

The test results for the MS2 bacteriophage aerosols are shown in Fig. 3. Specifically, Fig. 3A shows the effect of the MS2 virus concentration on the size distribution of the aerosolized particles at an AFR of 2 L/min for Case 3 (τ = 7.16 s). The total number concentration increased when the MS2 virus concentration increased. Figure 3B shows that an increase in the residence time leads to a decrease in the number concentration of aerosolized particles at a constant AFR of 2 L/min. However, after a residence time of 7.16 s, the number concentration of the aerosolized particles remained constant (Fig. 3C), thereby indicating that this corresponds to a sufficient diffusion drying time that maximizes droplet removal at an AFR of 2 L/min.

While the SMPS data (Fig. 3C) showed that the total number concentration varied with residence time, it is interesting to note that the plaque assay results remained constant with an average of 2.72 × 10^6^ plaque forming units per milliliter (PFU/mL) for each case (Fig. 3D). Virus aerosols were sampled for the plaque assay through the experiment setting shown in path B of Fig. 1. A gelatin filter was used. The virus concentration of 2.72 × 10^6^ PFU/mL on the gelatin filter corresponded to a total virus number of 1.8 × 10^9^ on the gelatin filter, which was obtained from the collection of aerosolized MS2 virus particles per 1 cm^3^ of air volume, at an AFR of 2 L/min and sampling time of 5 min (1.8 × 10^5^ virus particles/cm^3^ for τ > 7.16 s, see Fig. 3C).

The collection efficiency of the filter remained constant at 97% for all the cases. However, for τ < 7.16 s, the concentration of 2.72 × 10^6^ PFU/mL corresponded to a total virus number exceeding 1.8 × 10^9^. This was because the SMPS data for τ < 7.16 s were affected by non-dried water droplets as opposed to virus aerosols alone. The SMPS data in Fig. 3C were used to calculate error using Eq. (5). The following equation was used to quantify the effect of water droplets on the total number concentration of virus aerosol.

5 $$\mathrm{Error}(\%)=\frac{\mathrm{N}-{\mathrm{N}}_{{\max}}}{{\mathrm{N}}_{{\max}}}\times 100$$

where N denotes the total number concentration for a residence time $\uptau$, and ${\mathrm{N}}_{{\max}}$ denotes the total number concentration for the time required for maximum droplet removal.

For example, when τ = 0 s and total number concentration is 4.55 × 10^5^ cm^−3^ (N), the error in the total number concentration measurement is approximately 151% when considering 1.8 × 10^5^ cm^−3^ (${\mathrm{N}}_{{\max}}$) at a sufficient drying time (τ = 7.16 s). Similarly, for τ = 2.6 s and a total number concentration of 2.7 × 10^5^ cm^−3^, the error decreases to approximately 50%.

## Discussion

In recent years, there has been an increasing interest in air monitoring and aerosol measurements. Specifically, numerous studies have examined airborne virus transmission routes during the current COVID-19 pandemic as well as continuously emerging virus mutations, which facilitate virus infection even at low concentrations^11–14^. Additionally, the performance of airborne virus processing devices (e.g., devices for virus sampling and filtration) is evaluated via their collection efficiencies, which are calculated using the aerosolized virus number concentration. This can be measured upstream and downstream of a device by using particle counters such as SMPS^15,16^. Given that many of the routine laboratory procedures used to study and process virus samples (sample de-capping, pipetting, centrifugation, and mixing) exhibit high potential for producing aerosols^13,17,18^, it is important to adopt strict safety precautions when artificially aerosolizing virus particles using atomizers. Hence, it is preferable to generate aerosolized viruses at low concentrations to reduce health risks^16,19^ and mimic the actual environment in which airborne virus particles are present as low concentrations^20,21^. Several studies indicate that SMPS results are affected by particle shape and morphology and by the upper and lower size limits set by the instrument^5,6,22^. It is well known that the performance of SMPS may vary with respect to each particle size^23^. Most atomization processes generate virus particles of interest along with nanosized water droplets that can be mistakenly detected by an SMPS as virus particles. Along with Fig. 2, additional experiments were carried out to confirm the existence of nano water droplets. The results are presented in Supplementary materials as Figs. S1, S2, and S3. Thus, it is important to investigate size distributions and concentrations of these water droplets and evaluate their effect on overall measurements.

To the best of the authors’ knowledge, this is the first study to evaluate the importance of residence time in diffusion dryers and its effect on number concentration measurement. Furthermore, this is the first study to propose an empirical equation for determining the total number concentration of aerosolized particles. Moreover, our results indicated that nanosized droplets are generated at number concentrations ranging from 1.7 × 10^3^ to 1.2 × 10^6^ cm^−3^ when using different AFRs and residence times (Fig. 2, Table S1). The findings suggest that residence time significantly affects the accuracy of the aerosolized particle measurements because the plaque assay analysis reveals that the average PFU/mL number for all tested residence times remained constant (2.72 × 10^6^ PFU/mL), whereas the total number concentration measured by the SMPS decreased with increasing τ and was maintained constant (1.8 × 10^5^ virus particles per 1 cm^3^ of air volume) for τ > 7.16 s (Fig. 3C).

**Figure 4:**
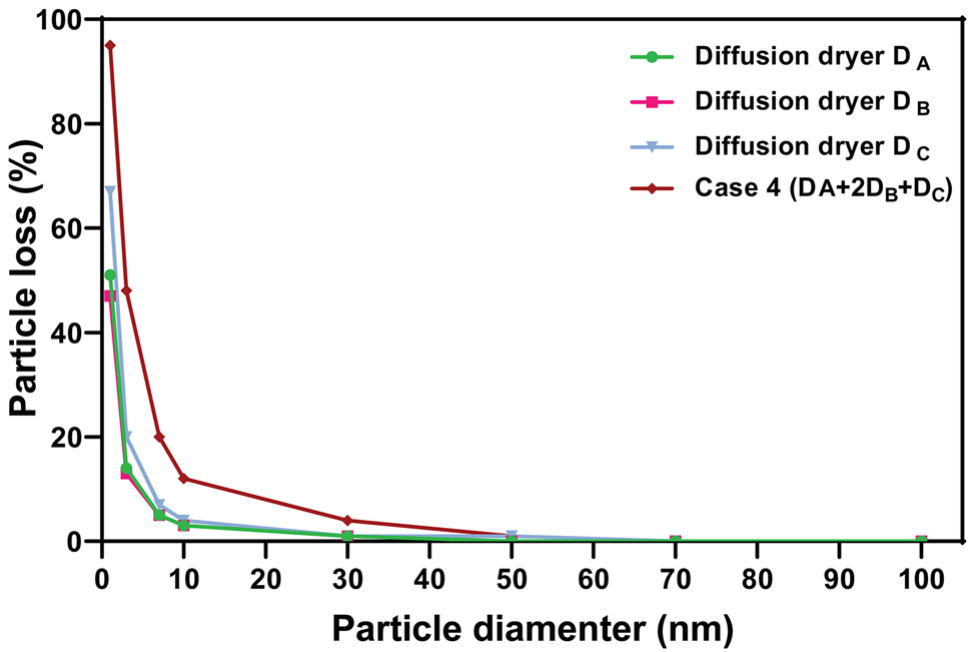
Particle loss per particle diameter for 2 L/min air flow rate.

Whether diffusion loss could affect the decrease in nanoparticle concentration, the following Gormley and Kennedy equation (Eq. 6)^24^ was investigated. The penetration efficiencies (${\eta }_{D}$) of particles passing through diffusion dryers were calculated, as follows,

6a $${\eta }_{D}=1-2.56{\xi }^{\left(\frac{2}{3}\right)}+1.2\xi +0.177{\xi }^{\left(\frac{4}{3}\right)}\qquad (\mathrm{if}\; \upxi <0.02)$$

6b $${\eta }_{D}=0.819{e}^{-3.657\xi }+0.097{e}^{-22.3\xi }+0.032{e}^{-57\xi }\qquad (\mathrm{if}\; \upxi >0.02)$$

where $\xi$ is the dimensionless parameter,

7 $$\xi =\frac{4D\tau }{{{w}_{i}}^{2}}$$

where D is the diffusion coefficient^25^,

8 $$D=\frac{kT{C}_{C}}{3\pi \mu {d}_{p}}$$

where ${d}_{p}$ is the particle diameter, $\mu$ the viscosity of air,* k* is the Boltzmann constant, ${C}_{C}$ the slip correction factor and T the absolute temperature. The calculations were made based on diffusion dryers’ characteristics (Table 1), AFRs ranging from 2–5 L/min, and particle sizes ranging from 1 to 100 nm. The results are presented in Fig. S4. The diffusional particle loss (L_D_) of each diffusion dryer for 2 L/min AFR was calculated with the following equation. The results are presented in Fig. 4.

9 $${L}_{D}=1-{\eta }_{D}$$

The diffusional particle loss of Case 4 was less than 10% for 20 nm particle size and decreased to almost zero with the increase of diameter (see Fig. 4). Considering the results of Fig. 4 and size distributions of aerosolized nanoparticles (Figs. 2 and 3), it is assumed that the decrease in the total number concentration of MS2 virus shown in Fig. 3C was not due to diffusional loss but solely due to the evaporation of non-virus-carrying droplets.

The percentage of error calculated using Eq. (5) indicated that when τ = 0 s, the error in the total number concentration measurement was approximately 151%. Similarly, when τ = 2.6 s, the error decreased to approximately 50%. This error was calculated as 0% for τ > 7.16 s.

Therefore, to increase the data accuracy from number concentration measurements and reduce uncertainties, we suggest using sufficient diffusion residence times, thus realizing maximum droplet removal when using the atomization method. Furthermore, another approach can be utilized, which involves diluting the air stream exiting from the atomizer with a clean air stream, thus realizing a lower air RH. In this study, the effect of nano droplet generation and diffusion drying was investigated for pressure type atomization. For other methods such as electrostatic and ultrasonic/sonic (whistle)-based atomization, further study is needed to explore the droplet generation characteristics and appropriate diffusion drying conditions.

## Methods

### Generation of nanosized water droplet aerosols

Sterile ultrapure DNase/RNase-Free, Protease-Free deionized (DI) water (W4502, Sigma-Aldrich, USA) (50 mL) was poured into a conventional atomizer (Single-Jet Atomizer 9302, TSI Inc., USA) and aerosolized according to the manufacturer’s protocols. Clean air with an AFR of 2–5 L/min was directed into the atomizer. The AFR was controlled using a mass flow meter (MFM, Mass Flow Meter 4140, TSI Inc.). Subsequently, the moisture and charge of the aerosol particles were removed via a series of diffusion dryers and an aerosol charge neutralizer (Soft X-ray charger 4530, HCT, Korea). A freshly replaced adsorbent was used in each experiment (chemically pure grade silica gel Blue, 7510–1400, Daejung Co., South Korea).

The size distribution of the aerosolized water droplets was measured using an SMPS (TSI model 3936L76, USA). The SMPS consists of a classifier controller (3080, TSI, USA), condensation particle counter (CPC, TSI model 3776, USA), differential mobility analyzer (DMA, TSI model 3085, USA), and an aerosol charge neutralizer (4530, HCT, Korea). The RH for each experiment was measured using a capacitive humidity sensor (Testo 0635 1535 three in one probe, DE). A schematic of the experimental design is shown in Fig. 1 (path A).

### Generation of virus aerosols

The experiments described above were repeated using an atomizer containing a virus solution. The MS2 bacteriophage was selected because it is not pathogenic to humans and is similar in structure and function to many human enteroviruses^26^. The MS2 bacteriophage uses *Escherichia coli* as a host for reproduction. An aliquot of the MS2 bacteriophage virus (ATCC 15597-B1, USA) solution (10–100 μL) was added to 50 mL of sterilized DI water. Different concentrations of MS2 bacteriophage (5.14 × 10^3^–4.11 × 10^4^ PFU/mL) were tested. Each virus solution was transferred to a TSI atomizer. The air was then directed into the atomizer at a flow rate of 2 L/min, thereby creating an aerosolized mixture of water and viruses. Subsequently, the air flow passed through a series of diffusion dryers for pure water droplet removal.

### Number concentrations and size distribution measurements

The size distribution and total number concentration of the aerosolized particles were measured via SMPS. The aerosolized particles were sampled on a Sartorius gelatin filter (12602-025-ALK, Sartorius Stedim Biotech, DE) with a diameter of 25 mm using an SKC Button aerosol sampler (SKC Inc., USA). The sampling time was set to 5 min, and the experiments were conducted in triplicate. Immediately after sampling, the gelatin filter was removed from the SKC Button sampler and immersed in 10 mL of sterile DI water for virus recovery, and the virus sample solution was used for plaque assay analysis. A schematic of the experimental design is shown in Fig. 1 (path B).

### Plaque assay analysis

In the study, PFU/mL concentrations were obtained after serial dilution of the sampled virus particles. Briefly, each gelatin filter containing the sampled virus was dissolved in 10 mL of DI water. Next, 0.1 mL of the serially diluted virus solutions were mixed with 0.3 mL of the *E. coli* C-3000 (ATCC 15597) hosted bacteria and 29 mL of tryptic soy agar (TSA, Difco, Korea). The mixture was poured into a Petri dish and incubated at 37 °C under aerobic conditions in an atmosphere of 95% RH for 24 h. The viral PFU number was then counted. Finally, the following equation was used to calculate the concentration of infectious virus particles (PFU/mL):

10 $$\frac{\mathrm{PFU}}{\mathrm{mL}}=\frac{{\mathrm{N}}_{{\rm p}}}{{10}^{-\mathrm{D}}\times \mathrm{ V}}$$

where N_p_ denotes the average number of plaques counted, D denotes the number of dilutions, and V denotes the volume of the virus solution. Further details are provided in Fig. S5 in Supplementary Material.

### Statistical analysis

All experiments were performed in triplicate, as indicated in the figure legends. Data are expressed as the mean ± standard error of the mean. Statistical analyses were performed using GraphPad Prism version 8.

## Supplementary Information

Supplementary Information.
