# Supplementary material for: Effects of nanosized water droplet generation on number concentration measurement of virus aerosols when using an airblast atomizer
Source: Sci Rep. 2022 Apr 21;12:6546. doi: 10.1038/s41598-022-10440-4 (PMC9022418; doi:10.1038/s41598-022-10440-4)
Supplement: Supplementary file 1 — Supplementary Information. [file 41598_2022_10440_MOESM1_ESM.pdf]

Supplementary Materials for

Effects of nanosized water droplet generation on number concentration measurement of virus aerosols when using an airblast atomizer

Milad Massoudifarid[1,‡], Amin Piri[1,‡], and Jungho Hwang[1,*]

[1] Department of Mechanical Engineering

Yonsei University, Seoul 03722, Republic of Korea

‡ These authors contributed equally to the work.

Corresponding author: Jungho Hwang, Department of Mechanical Engineering, Yonsei University, Seoul 120-749, Republic of Korea. E-mail: hwangjh@yonsei.ac.kr; Tel.: +82-2-2123-2821; Fax: +82-2-312-2821

Table S1. Characteristics of the aerosolized water droplets and relative humidity under different experimental conditions.

Air flow rate (L/min)	Residence Time (s)	Total number concentration (cm^{-3})	Mode (nm)	Geometric mean diameter (nm)	Relative Humidity (%)
	0	150302	55.95	48.63	91.1
	2.64	58966	45.78	41.47	62
**2**	4.90	29723	47.92	42.67	40.1
	7.16	15736	55.4	42.03	30.1
	9.70	1733	40	29	17.6
	0	640880	52.65	49.23	95.6
	1.76	243616	44	42.49	72
**3**	3.27	67688	39.05	33.36	47.7
	4.77	34260	43	35.14	36.6
	6.47	6236	33.4	30	22
	0	1001997	52.65	48.44	96.9
	1.13	327184	41.92	41.22	77
**4**	2.45	83181	29.97	28.2	52
	3.58	71716	37.74	32.41	41.1
	4.85	9283	26.89	25.77	27
	0	1156366	50.82	46.46	97.9
	1	306039	38.11	37.39	79.6
**5**	1.96	91851	28.24	26.59	54.6
	2.86	94981	34.23	29.09	44
	3.88	13477	23.8	24.1	32

To provide sufficient evidence regarding the accuracy of generation of nana droplets in Fig. 2, we performed the following experiments:

(1) Investigation of possible contaminations in the compressed clean air supply system

All experiments were performed in a biosafety level 2 approved facility equipped with clean aerosol booths to avoid contaminations. Moreover, to check traces of contamination or impurities in our compressed clean air supply system, the air stream was directly connected to the SMPS, and the particles size distributions was measured. The data showed there were no significant particles (Fig. S3). Also, prior to each experiment, the atomizer, and other components such as diffusion dryers were fully rinsed and dried with compressed dry air to avoid any nano particles dried on the wall of these devices. Moreover, the silica gels as well as all the connecting tubes were replaced with new ones prior to each experiment. Thus, there could not have been any impurities in air steam during our experiments.

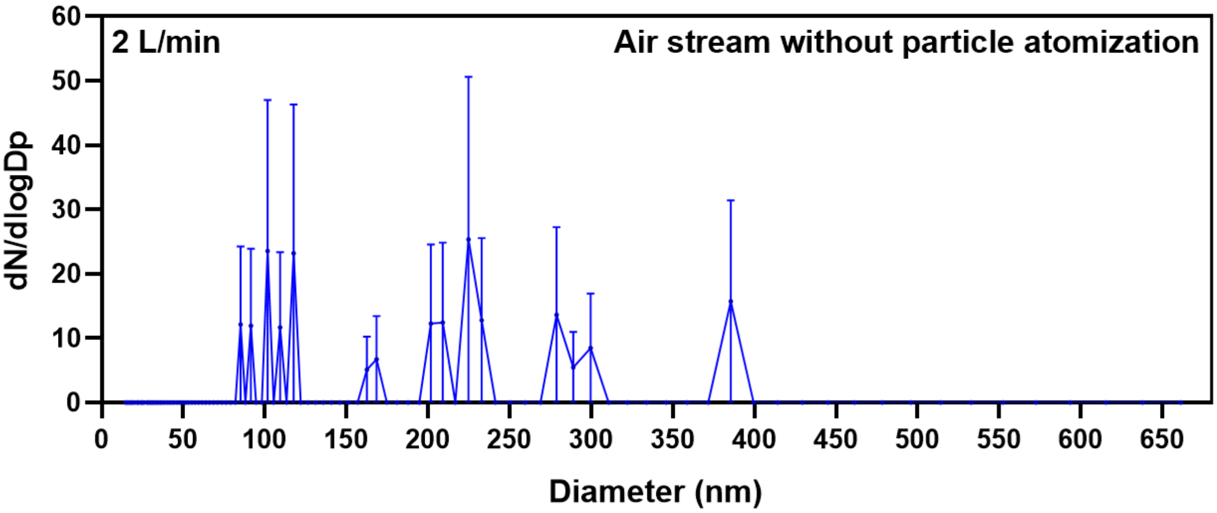

Fig. S1 Size distribution and particle number concentration for nano residuals from atomizer, tubes, and diffusion dryers

(2) Confirming the existence of the nano water droplets in the air stream

In a separate experiment, **to confirm the existence of the nano water droplets**, the air stream was directed into an airtight chamber containing a RH meter (Fig. S4). Then the chamber was closed and the alterations in air RH was monitored as time passed.

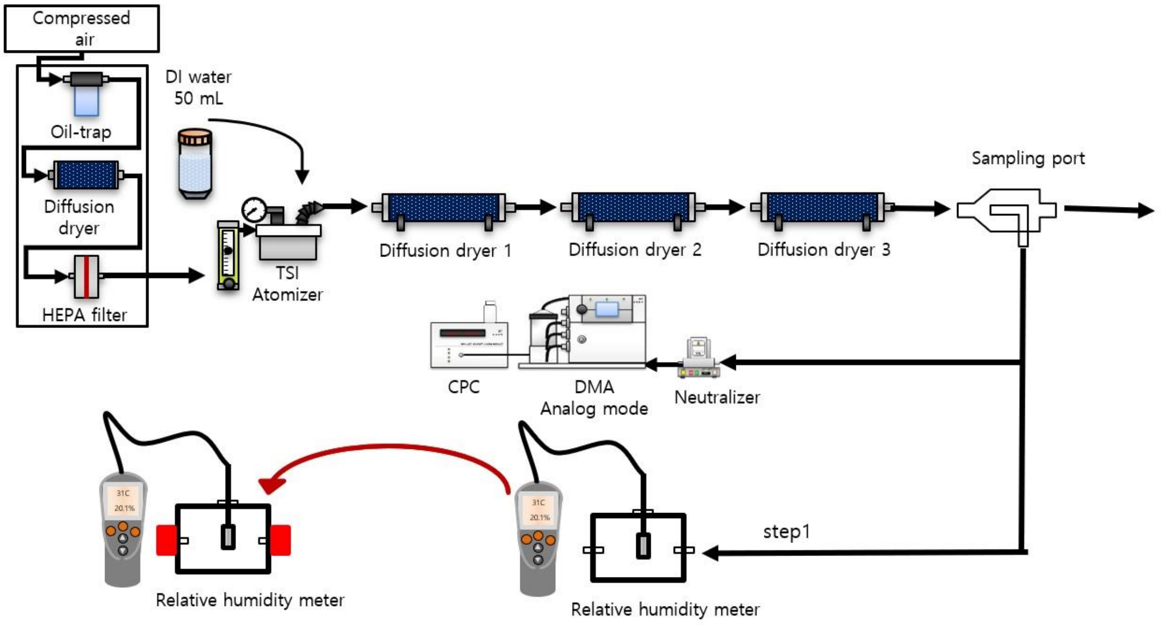

Fig. S2 Schematic representation of the tests for confirming the existence of water droplets in the air stream

Experiment details: DI water was used as the atomizing solution and the nano water droplets were aerosolized with an air flow rate of 2 L/min. Then, the air stream passed through a series of diffusion dryers and their size distribution was measured using a SMPS while the air RH was measured using an air RH meter. When the air RH value inside the duct became stable, both inlet and outlet of the chamber duct was sealed. Next, the changes in RH were monitored for a duration of 35 min. Considering that if the particles by measured SMPS were water droplets, in that case the existence of water droplets would lead to increase in RH over time.

Result: As predicted, in a period of 35 min, the air RH has approximately increased from 20 to 30% (Fig. S5). This clearly shows that: (1) the measured particles indeed were water droplets since their evaporation had led to the increase in RH value, (2) the aerosolized nano water droplets do not evaporate immediately after exiting the atomizer and the gradually evaporated in during a long period of 35 min or higher.

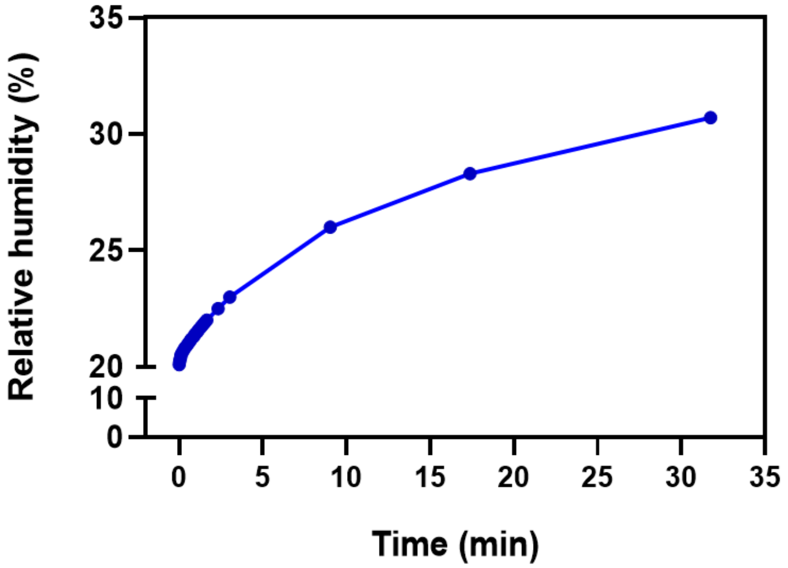

Fig. S3 changes of Relative Humidity inside the sealed chamber with the time, %

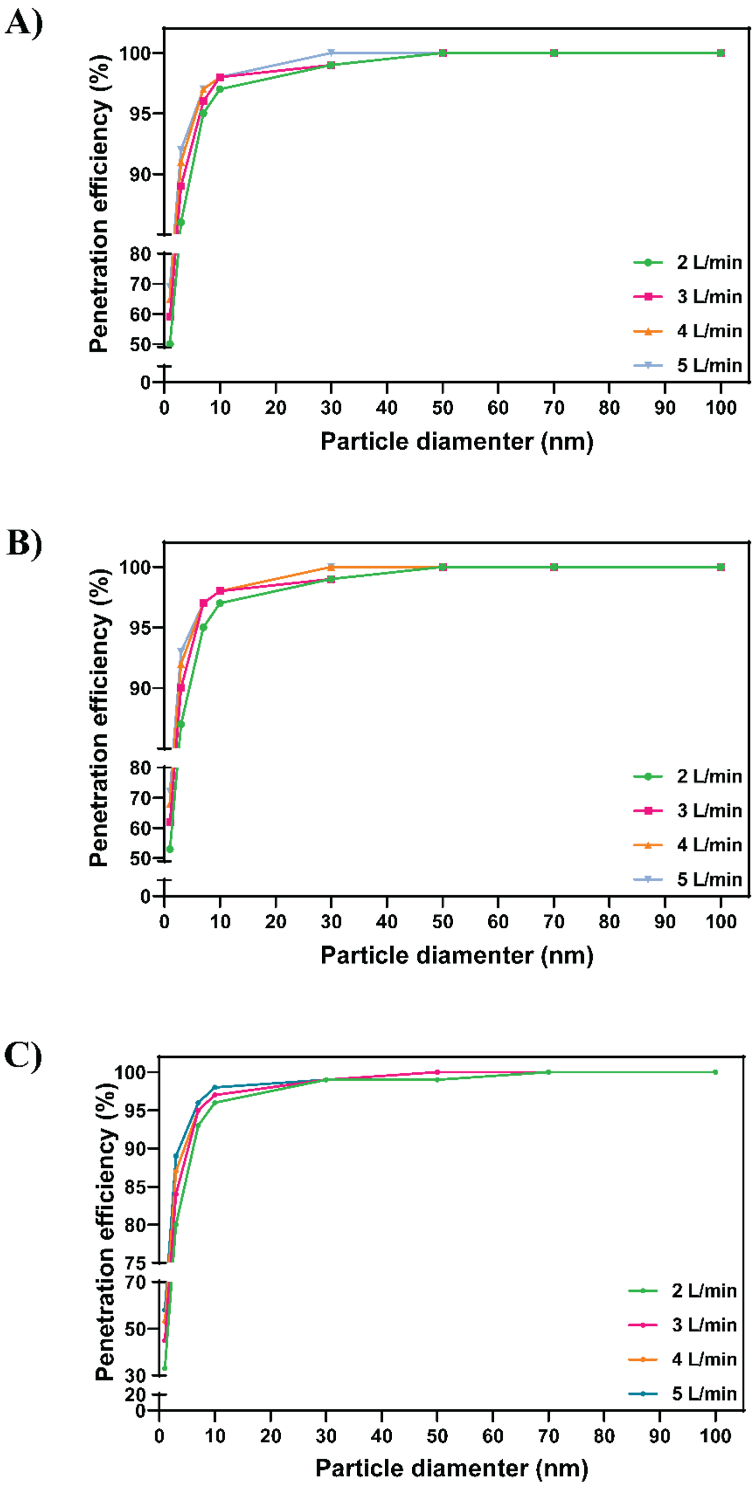

Fig. S4 Penetration efficiency of particles through diffusion dryers for different flow rates. A) Diffusion dryer type D_A B) Diffusion dryer type D_B C) Diffusion dryer type D_C

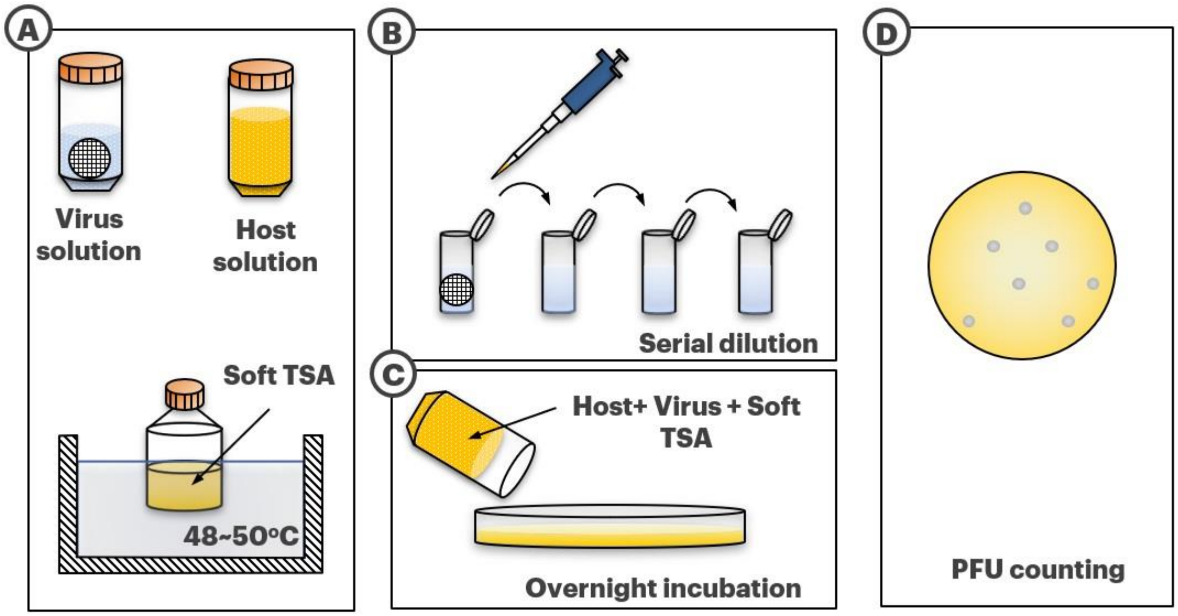

Fig. S5 Schematic representation of the plaque assay test using the aerosolized virus.
